# Association between fibrinogen levels and stroke-associated pneumonia in acute ischemic stroke patients

**DOI:** 10.1186/s12883-024-03752-7

**Published:** 2024-07-24

**Authors:** Xiaoqiang Li, Hui Du, Zhibin Song, Meiqi, Guifeng Zhang, Suhua Yuan, Yuanfeng, Hui Wang

**Affiliations:** 1 Department of Neurology, Xiaolan People’s Hospital of Zhongshan, No. 65, Jucheng Rd. Xiaolan Dist, Zhongshan, Guangdong Prov 528415 P.R. China; 2 Department of Blood Transfusion, Xiaolan People’s Hospital of Zhongshan, Zhongshan, Guangdong China; 3 Medical Records Room, Xiaolan People’s Hospital of Zhongshan, Zhongshan, Guangdong China; 4 Southern Medical University, Guangzhou, Guangdong China

**Keywords:** Stroke-associated pneumonia, Fibrinogen, Acute ischemic stroke, Pneumonia

## Abstract

**Purpose:**

Prior research had indicated a relationship between fibrinogen and stroke-associated pneumonia (SAP), yet the nature of this relationship had not been thoroughly investigated. Therefore, this study was designed to elucidate the prognostic value of fibrinogen levels in forecasting the occurrence of SAP among patients with acute ischemic stroke (AIS).

**Patients and methods:**

In this retrospective cross-sectional analysis, we included 1092 patients who had experienced AIS and were admitted to our facility within 72 h of the onset of their symptoms. Based on the SAP diagnostic criteria, patients were classified into two groups: SAP and non-SAP. The correlation between serum fibrinogen concentration and SAP was examined using univariate analysis. Curve fitting and multivariable logistic regression model were utilized for statistical evaluation.

**Results:**

Out of the ischemic stroke patients included in the study, SAP was identified in 112 (10.26%) patients. A direct correlation was observed between fibrinogen levels and the incidence of SAP. An increase in fibrinogen levels corresponded with a heightened incidence of SAP. Multivariable logistic regression revealed a significant positive association between fibrinogen levels and SAP incidence (OR = 1.53, 95% confidence interval [CI]: 1.18, 1.99)).

**Conclusion:**

A linear relationship between serum fibrinogen levels and the incidence of SAP in ischemic stroke patients was shown. The serum fibrinogen levels were positively and linearly correlated to SAP risk.

## Introduction

Stroke stands as a significant global health concern, with the annual number of strokes and deaths due to stroke increasing substantially from 1990 to 2019 [1, 2]. In China, the burden of stroke is particularly severe, with an estimated 3.94 million new stroke cases and 2.19 million stroke deaths in 2020 [3]. An increasing body of evidence identifies SAP as a common and severe medical complication post-stroke, with prevalence rates varying between 3.2% and 56.6% in different sitting [4–7]. SAP often results in poor functional outcomes following an AIS, leading to increased mortality, morbidity, and extended hospital stays [7–10]. Therefore, the early detection and effective prevention of SAP are of paramount importance, superseding its treatment.

Fibrinogen, a final product of the coagulation cascade, is also a classic acute phase reactant and a potent modulator of inflammatory processes [11]. Numerous studies indicates that hyperfibrinogenemia plays a crucial role in AIS pathogenesis and is associated with adverse outcomes, including higher in-hospital mortality, impaired cognitive functions, and recurrent ischemic events [12–16]. Although it is known that fibrinogen participates in chronic low-grade inflammation, further investigation is required to elucidate its capacity to indicate the inflammatory status in individuals with stroke [17].

The relationship between fibrinogen levels and SAP in AIS patients is currently not well-defined. This study aims to elucidate the association between serum fibrinogen levels and SAP in AIS patients. While some research has indicated a correlation between serum fibrinogen and SAP, the linear and non-linear relationships between fibrinogen levels and SAP in AIS patients have not been thoroughly investigated [18, 19]. Our study seeks to determine whether fibrinogen is independently associated with SAP and to explore the intricate relationships between fibrinogen levels and SAP in AIS patients from China. Our findings may contribute to improving AIS prevention and treatment strategies within the Chinese demographic.

## Methods

### Research participants

In a retrospective cross-sectional study, we investigated the association between fibrinogen levels and SAP in patients with AIS. Data from AIS patients were systematically and consecutively gathered from the Endocrinology Department of Xiaolan People’s Hospital, Zhongshan, China. To ensure participant privacy, the collected data were anonymized and stored within the hospital’s electronic medical record system. The retrospective cross-sectional nature of this study helps to mitigate potential selection and observation biases.

This retrospective study was conducted at Xiaolan People’s Hospital of Zhongshan with the following inclusion criteria: (1) Diagnosis of ischemic stroke in patients, based on World Health Organization guidelines, corroborated by cranial MRI or CT scans alongside corresponding clinical symptoms [20]; (2) Admission of patients within a 72-hour window following stroke onset; (3) Patients aged over 18 years; (4) Complete clinical information, including fibrinogen and SAP records.

Participants were excluded based on the following criteria: (1) Existence of pneumonia or active infections prior to hospital admission; (2) History of blood disorders, other central nervous system injuries, or current immunosuppressive therapy; (3) Transient ischemic attack (TIA) or Cerebral hemorrhage; (4) Use of prophylactic antibiotics. The Ethics Committee of Xiaolan People’s Hospital of Zhongshan approved this study, and due to its observational and retrospective nature, the requirement for informed consent was waived.

**Fig. 1:**
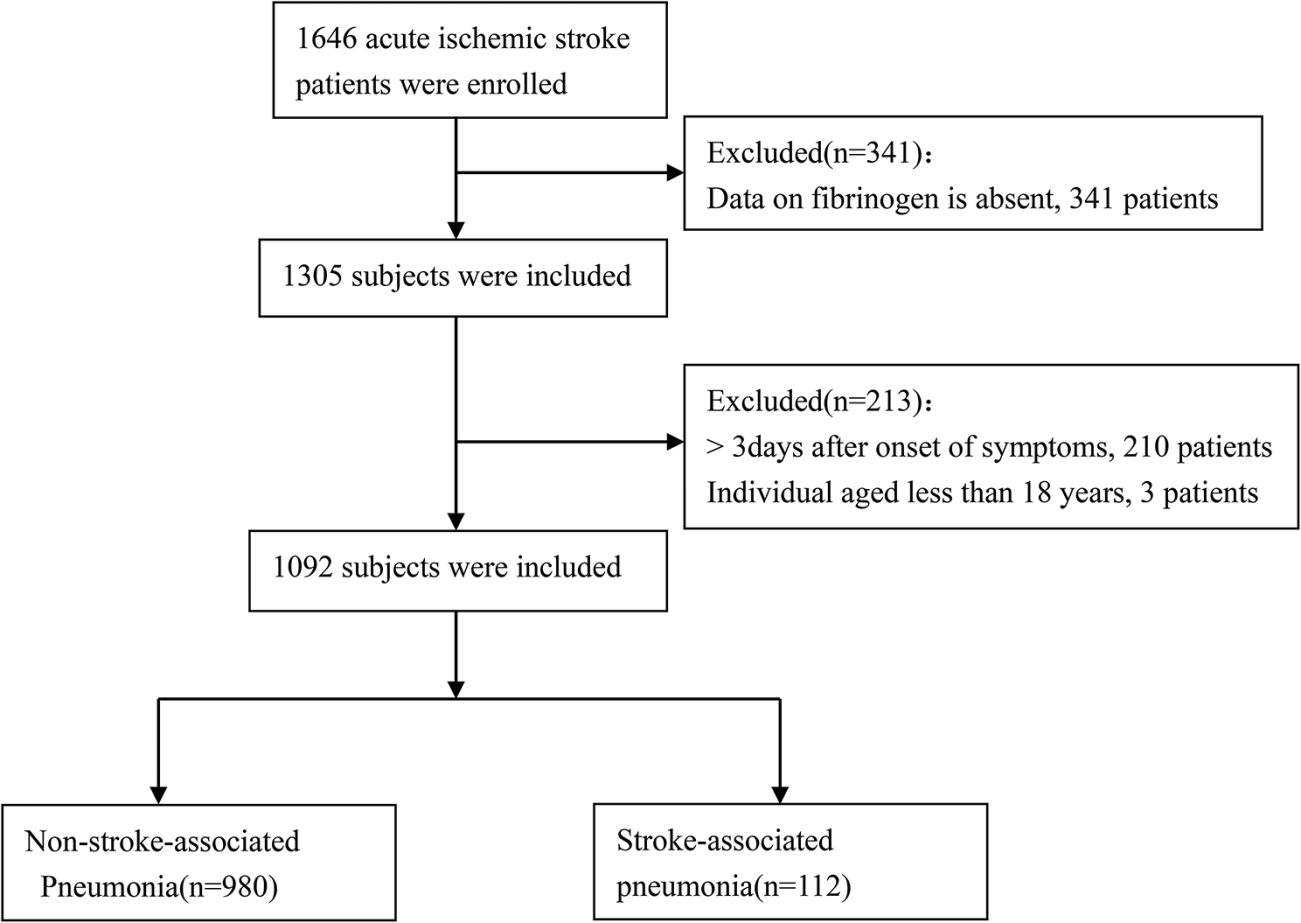
Flow chart visualizing the patient selection process

Whatmore, Outliers(14 of 1092) in fibrinogen data were appropriately addressed [21, 22]. According to these criteria, a total of 1092 consecutive AIS patients were retrospectively included in this study from October 2019 to November 2022 (Fig. 1).

### SAP diagnosis

Diagnosis of SAP adhered to the standardized criteria from the 2015 consensus [23], utilizing clinical assessments and laboratory tests, with validation by retrospective sputum culture analyses and chest CT evaluations documented in medical records.

### Data acquisition

From each patient’s electronic medical record, the ensuing demographic and clinical information was gathered: sex, age, diagnosis, length of hospital stay, comorbidities (diabetes, hypertension, atrial fibrillation), venous thrombolysis, and SAP. Laboratory examinations at admission were also recorded, including triacylglycerol (TG), High-density lipoprotein cholesterol (HDL-C), low-density lipoprotein cholesterol (LDL-C), total cholesterol (TC), blood glucose, white blood cell (WBC), neutrophils, lymphocytes, and fibrinogen levels. Elbow venous blood (5 mL) was drawn from all participants after a minimum of 12-hour fasting. Blood parameters including triglyceride (TG), total cholesterol (TC), high/low-density lipoprotein cholesterol (H/LDL). Serum fibrinogen concentrations were measured immediately following admission through these samples and analyzed using an automatic coagulation analyzer (CS-5100; Sysmex, Kobe Japan).

### Statistical evaluation

Continuous variables were presented as mean ± standard deviation and count data as the number of cases (%). For comparing continuous variables, the two-sample t-test or Wilcoxon rank-sum test was employed, whereas the χ2 test and Fisher’s exact test were utilized for categorical variables. Curve fitting analysis was performed to investigate the relationship between fibrinogen levels and the incidence of SAP. This method allows for the exploration of both linear and non-linear relationships. Univariate and multivariate Cox proportional risk models were used to evaluate the relationship between fibrinogen level and SAP risk. Three models were used: model 1 (crude model), model 2 (adjusted for age and sex), and model 3 (adjusted for multiple factors including sex, age, length of hospital stay, comorbidities, venous thrombolysis, various blood parameters). Furthermore, curve fitting was utilized to explore the relationship between fibrinogen level and incidence of comorbiditiesa in AIS patients. A *p*-value of < 0.05 was considered statistically significant. All analyses were conducted using the statistical software R (https://www.R-project.org, The R Foundation) and EmpowerStats (http://www.empowerstats.com, X&Y Solutions, Inc., Boston, MA, USA).

## Result

### Participant characteristics

This study encompassed a total of 1092 AIS patients. Among these, 112 patients (10.26%) were diagnosed with SAP, while 980 patients (89.74%) were categorized as non-SAP.

A comparative analysis of clinical and laboratory parameters between the SAP and non-SAP groups was presented in Table 1. The SAP group was significantly older, had longer hospital stays, elevated fibrinogen, higher WBC and neutrophil counts, HDL-C, atrial fibrillation and venous thrombolysis compared to the non-SAP group (*P* < 0.05). However, the non-SAP group also exhibited elevated lymphocytes, TG compared to the non-SAP group (Table 1). There were no significant differences in the remaining parameters (*P >* 0.05).

**Table 1:** Baseline characteristics of participants according to SAP

	Non-SAP (980)	SAP (112)	*P*-value
Sex(male)	674 (68.78%)	73 (65.18%)	0.438
Age, years	61.36 ± 12.51	67.87 ± 14.37	< 0.001
Length of hospital stay, days	9.46 ± 5.11	12.35 ± 6.93	< 0.001
Fibrinogen, g/L	3.09 ± 0.81	3.43 ± 1.11	< 0.001
WBC, 10^9/L	8.24 ± 2.73	10.26 ± 4.04	< 0.001
Lymphocytes, 10^9/L	1.65 ± 0.69	1.25 ± 0.60	< 0.001
Neutrophils, 10^9/L	5.96 ± 2.66	8.30 ± 3.90	< 0.001
TG, mmol/L	1.75 ± 1.28	1.42 ± 0.90	0.013
HDL-C, mmol/L	1.09 ± 0.29	1.15 ± 0.36	0.049
LDL-C, mmol/L	3.11 ± 0.98	2.99 ± 1.20	0.254
TC, mmol/L	4.68 ± 1.12	4.51 ± 1.30	0.152
Blood glucose, mmol/L	8.43 ± 4.58	8.39 ± 4.38	0.922
Diabetes	357 (36.43%)	34 (30.36%)	0.204
Hypertension	824 (84.08%)	94 (83.93%)	0.967
Atrial fibrillation	52 (5.31%)	24 (21.43%)	< 0.001
Venous thrombolysis	133 (13.57%)	40 (35.71%)	< 0.001

*Notes* Continuous data are shown as mean ± SD (normal distribution) or median (quartile) (skewed distribution). Categorical data are shown as n (%). *Abbreviations* WBC: White blood cells. TG: Triglyceride, TC: Total cholesterol, LDL-C: Low-density lipoprotein cholesterol, HDL-C: High-density lipoprotein cholesterol, SAP: stroke-associated pneumonia

Table 1 Baseline characteristics of participants according to SAP.

Relationship between fibrinogen levels and incidence of SAP in AIS patients.

**Fig. 2:**
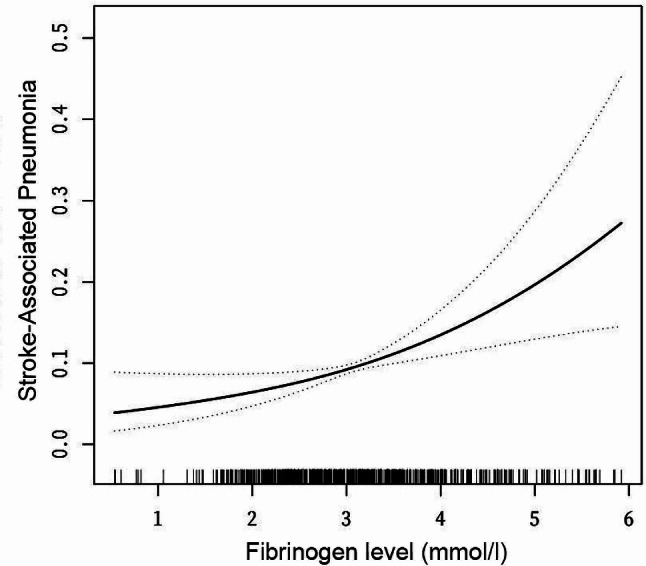
The relationship between fibrinogen Level and the incidence of SAP. Solid line represents the smooth curve fit between variables. Dotted line represents the 95% of confidence interval from the fit. All adjusted for: sex and age; length of hospital stay, diabetes, hypertension, atrial fibrillation; venous thrombolysis; TG, LDL-C and total cholesterol, HDL-C, blood glucose, lymphocytes, WBC

We applied curve fitting to investigate the relationship between fibrinogen levels and the incidence of SAP. Figure 2 illustrated the results of this analysis. The graph showed a positive trend between fibrinogen concentration and SAP incidence. The relationship appeared to be linear, with SAP incidence increasing as fibrinogen levels rose.

### Univariable analysis of the relationship between covariates and SAP

To identify the factors associated with the incidence of SAP, we conducted a univariate analysis (Table 2). Without adjusting for any confounding variables, the results of the univariate analysis revealed that fibrinogen levels (OR = 1.15, 95% CI: 1.25, 1.92), age (OR = 1.04, 95% CI: 1.03, 1.06), length of hospital stay (OR = 1.08, 95% CI: 1.05, 1.12), WBC (OR = 1.21, 95% CI: 1.14, 1.28), lymphocytes (OR = 0.32, 95% CI: 0.21, 0.47), neutrophils (OR = 1.24, 95% CI: 1.17, 1.32), TG (OR = 0.70, 95% CI: 0.54, 0.92), HDL-C (OR = 1.95, 95% CI: 1.00, 3.80), atrial fibrillation(OR = 4.87, 95% CI: 2.86, 8.28) and venous thrombolysis (OR = 3.54, 95% CI: 2.31, 5.43) were positively correlated with the incidence of SAP. In contrast, sex, blood glucose, LDL-C, diabetes, and hypertension were not found to be associated with SAP.

**Table 2:** Crude association to identify risk factors (or tertile) associated with SAP in AIS patients

	Statistics	OR(95% CI)	*P*-value
Sex (Male vs. Female)	(747vs 345)	1.18 (0.78, 1.78)	0.4383
Age (year)	62.02 ± 12.86	1.04 (1.03, 1.06)	< 0.0001
Low	343 (31.41%)	Ref	
Middle	350 (32.05%)	1.21 (0.68, 2.15)	0.5146
High	399 (36.54%)	2.51 (1.52, 4.15)	0.0003
Length of hospital stay	9.75 ± 5.39	1.08 (1.05, 1.12)	< 0.0001
Low	325 (29.76%)	Ref	
Middle	342 (31.32%)	1.04 (0.57, 1.89)	0.8993
High	425 (38.92%)	2.53 (1.53, 4.20)	0.0003
White blood cell	8.45 ± 2.96	1.21 (1.14, 1.28)	<0.0001
Low	334 (31.69%)	Ref	
Middle	366 (34.72%)	1.68 (0.93, 3.06)	0.0881
High	354 (33.59%)	3.58 (2.07, 6.21)	< 0.0001
Blood glucose	8.43 ± 4.56	1.00 (0.96, 1.04)	0.9221
Low	352 (33.08%)	Ref	
Middle	355 (33.36%)	1.46 (0.90, 2.38)	0.1231
High	357 (33.55%)	1.16 (0.70, 1.92)	0.5613
Lymphocytes	1.60 ± 0.70	0.32 (0.21, 0.47)	< 0.0001
Low	350 (33.21%)	Ref	
Middle	349 (33.11%)	0.46 (0.29, 0.74)	0.0013
High	355 (33.68%)	0.31 (0.18, 0.52)	< 0.0001
Neutrophils	6.20 ± 2.90	1.24 (1.17, 1.32)	< 0.0001
Low	351 (33.30%)	Ref	
Middle	351 (33.30%)	1.82 (0.96, 3.42)	0.0649
High	352 (33.40%)	4.83 (2.74, 8.53)	< 0.0001
TG	1.72 ± 1.25	0.70 (0.54, 0.92)	0.0099
Low	336 (32.72%)	Ref	
Middle	346 (33.69%)	0.66 (0.41, 1.09)	0.1055
High	345 (33.59%)	0.59 (0.36, 0.99)	0.0450
HDL-C	1.10 ± 0.30	1.95 (1.00, 3.80)	0.0493
Low	326 (31.87%)	Ref	
Middle	348 (34.02%)	1.00 (0.59, 1.72)	0.9883
High	349 (34.12%)	1.42 (0.85, 2.35)	0.1774
LDL-C	3.10 ± 1.01	0.88 (0.72, 1.09)	0.2534
Low	340 (33.24%)	Ref	
Middle	342 (33.43%)	0.40 (0.23, 0.69)	0.0010
High	341 (33.33%)	0.68 (0.43, 1.10)	0.1181
Fibrinogen	3.12 ± 0.85	1.55 (1.25, 1.92)	< 0.0001
Low	363 (33.24%)	Ref	
Middle	364 (33.33%)	1.15 (0.68, 1.96)	0.5977
High	365 (33.42%)	1.99 (1.22, 3.23)	0.0055
TC	4.66 ± 1.14	0.87 (0.72, 1.05)	0.1517
Low	342 (33.30%)	Ref	
Middle	342 (33.30%)	0.53 (0.32, 0.89)	0.0172
High	343 (33.40%)	0.65 (0.40, 1.06)	0.0842
Diabetes (without vs. with)	(701 vs.391)	0.76 (0.50, 1.16)	0.2053
Hypertension (without vs. with)	(174 vs. 918)	0.99 (0.58, 1.68)	0.8780
Atrial fibrillation (without vs. with)	(1016 vs. 76)	4.87 (2.86, 8.28)	< 0.0001
Venous thrombolysis (without vs. with)	(919 vs. 173)	3.54 (2.31, 5.43)	< 0.0001

*Notes* Bold values are considered statistically significant. *Abbreviations* OR: odds ratio, CI: confidence interval, WBC: White blood cells, TG: Triglyceride, TC: Total cholesterol, LDL-C: Low-density lipoprotein cholesterol, HDL-C: High-density lipoprotein cholesterol, SAP: stroke-associated pneumonia

### Multivariate analysis between the fibrinogen level and incidence of SAP

We conducted multivariate logistic regression analyses to examine the relationship between fibrinogen levels and SAP incidence. We used fibrinogen level as the independent variable, SAP risk as the dependent variable, and adjusted for a comprehensive range of variables: sex, age, length of hospital stay, and medical conditions including diabetes, hypertension, and atrial fibrillation. Additionally, it accounts for venous thrombolysis and a spectrum of biochemical and hematological parameters such as TG, HDL-C, TC, LDL-C, blood glucose, neutrophils, lymphocytes and WBC. Table 3 presented the results of the multivariate logistic regression analyses. In the unadjusted model (Model 1), the odds ratio (OR) for SAP associated with fibrinogen levels was 1.55 (95% CI: 1.25, 1.92; *p* < 0.001). After minimal adjustment (Model 2), the OR was 1.42 (95% CI: 1.14, 1.78; *p* = 0.0021). In the fully adjusted model (Model 3), which accounted for all covariates, the OR was 1.53 (95% CI: 1.18, 1.99; *p* = 0.0015). For sensitivity analysis, fibrinogen levels were additionally categorized into tertiles. In model III, compared with the T1 (reference group), the estimated increase of SAP in the T2 and T3 was 1.52 and 2.63, respectively.

**Table 3:** Relationship between fibrinogen level in different models of multivariate analysis

Variable	Model 1(OR, 95% CI, *P*)	model 2(OR, 95% CI, *P*)	model 3(OR, 95% CI, *P*)
Fibrinogen	1.55 (1.25, 1.92) < 0.0001	1.42 (1.14, 1.78) 0.0021	1.53 (1.18, 1.99) 0.0015
Fibrinogen (tertiles)			
T1	Ref	Ref	Ref
T2	1.15 (0.68, 1.96) 0.5977	1.02 (0.59, 1.74) 0.9533	1.52 (0.77, 3.00) 0.2238
T3	1.99 (1.22, 3.23) 0.0055	1.60 (0.97, 2.63) 0.0639	2.63 (1.36, 5.08) 0.0041

*Notes* Model 1: no variables are adjusted. Model 2 adjust for: sex and age. Model 3 adjust for: sex, age, length of hospital stay, diabetes, hypertension, atrial fibrillation, venous thrombolysis, TG, HDL-C, TC, LDL-C, blood glucose, neutrophils, lymphocytes and WBC

### Subgroup analysis

**Fig. 3:**
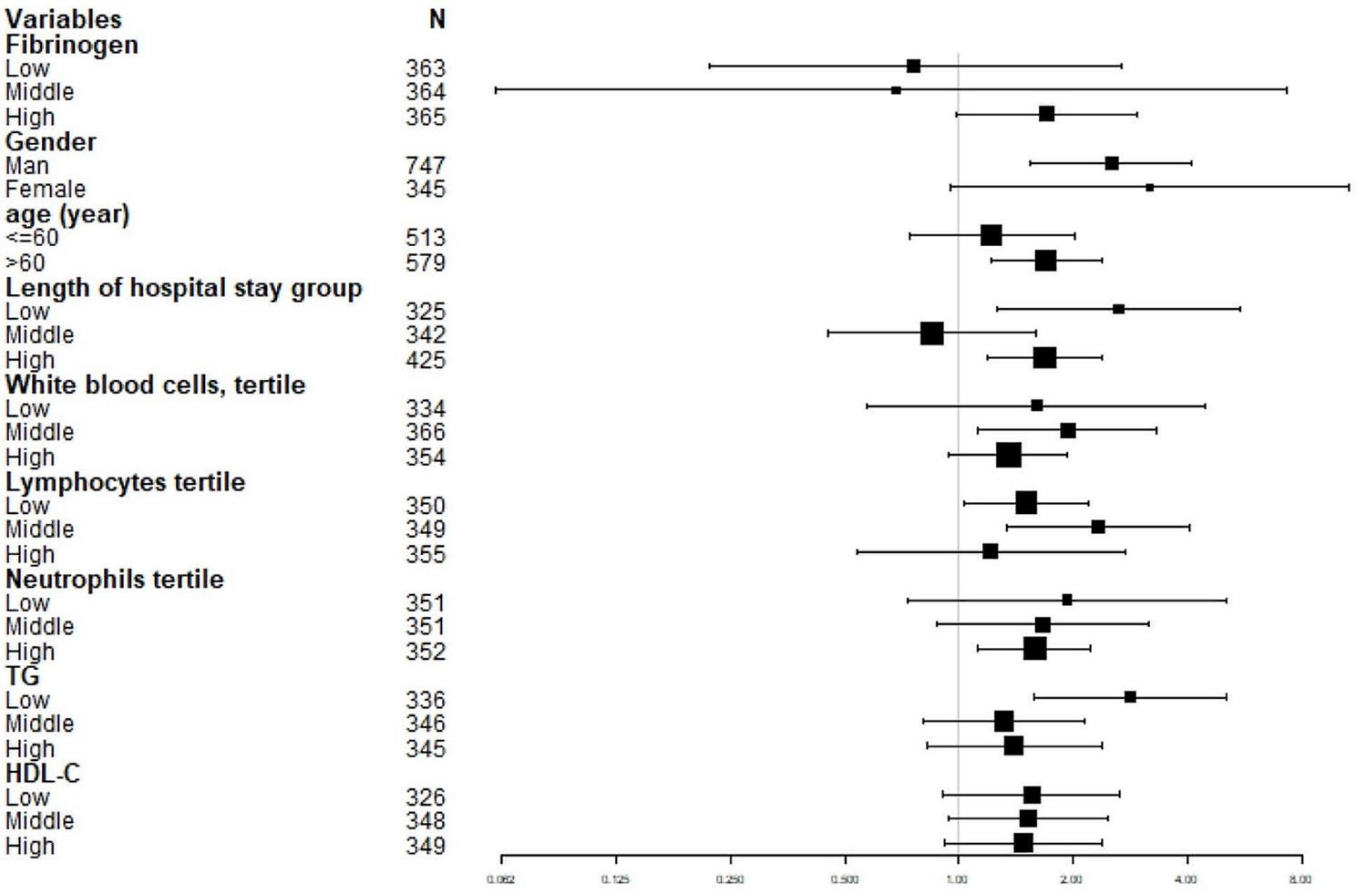
Forest plots for subgroup analysis of fibrinogen on SAP

To ensure the reliability of Table 3’s findings against confounders, we conducted stratified analyses by subgroups by covariables sex(man, woman), Age (≤ 60, >60), length of hospital stay group, WBC, lymphocytes, TG, and HDL-C group were stratified (Fig. 3). Figure 3; Table 3 revealed a highly consistent pattern.

## Discussion

In this retrospective analysis, we observed that patients with SAP demonstrated significantly elevated fibrinogen levels, advanced age, prolonged hospital stays, and increased counts of WBC, lymphocytes, and neutrophils at the time of admission. Moreover, a distinct independent association was established between heightened fibrinogen levels and the development of SAP, after adjusting for potential confounding factors. Notably, the incidence of SAP increased by 53% for each 1 mmol/l rise in fibrinogen levels. Subgroup analyses consistently supported this conclusion, underscoring the potential of fibrinogen as a predictive biomarker for assessing the risk of SAP. Consequently, fibrinogen may serve as a valuable and reliable indicator for the early identification of patients at heightened risk of developing SAP upon hospital admission.

In contrast to earlier research [24, 25], our study offers a comprehensive comparison of fibrinogen levels in patients with SAP compared to those without, establishing elevated fibrinogen as an independent risk factor for SAP. Our findings not only confirm the association between fibrinogen and SAP but also quantify this relationship, demonstrating a positive and linear correlation between serum fibrinogen levels and SAP risk. This quantification represents a significant advancement in our understanding of SAP risk factors. Our results align with those of Lin et al [25], who found that the fibrinogen-to-albumin ratio(FAR) was significantly higher in patients with SAP compared to those without. Their study identified FAR as an independent predictor of SAP, which complements our findings on fibrinogen levels alone. Similarly, Qiu et al [26] reported that the fibrinogen-prealbumin ratio (FPR) was independently associated with SAP, further supporting the role of fibrinogen in SAP development.

In our cohort, 112 patients (10.26%) were diagnosed with SAP, which is consistent with the prevalence reported in existing literature [25]. Our analysis identified several factors contributing to SAP, including age, duration of hospitalization, WBC count, lymphocyte levels, TG, HDL-C, neutrophil count, atrial fibrillation, and venous thrombolysis. These findings are in concordance with previous studies [27, 28]. While these risk factors have been previously recognized, our study is among the first to quantify the specific contribution of fibrinogen to SAP risk, thereby enhancing the precision of risk assessment in AIS patients. Interestingly, Luo et al [29] found that the neutrophil-to-lymphocyte ratio (NLR) and platelet-to-lymphocyte ratio (PLR) were independent predictors of pneumonia in patients with acute intracerebral hemorrhage. Although their study focused on a different type of stroke, it highlights the importance of inflammatory markers in predicting post-stroke infections. Furthermore, the study by Zu and Zhang [30] on infectious pneumonia in animal models demonstrated that fibrinogen levels were significantly elevated in pneumonia, supporting our findings in the context of SAP. Their study also revealed an imbalance in T helper 17 cells and regulatory T cells, suggesting potential mechanisms underlying the association between fibrinogen and SAP that warrant further investigation in human subjects.

Moreover, our research demonstrated that within a patient cohort characterized by diverse fibrinogen levels, varying genders, and different lengths of hospitalization, individuals exhibiting elevated fibrinogen levels exhibited a heightened susceptibility to SAP. This effect was evident in all subgroups considered and after careful adjustments. This consistency across subgroups strengthens the reliability of our findings and suggests that fibrinogen could serve as a universal biomarker for SAP risk, regardless of patient demographics or clinical characteristics.

The pathophysiological mechanisms underlying the association between fibrinogen and SAP are complex and multifaceted. While dysphagia and the associated risk of aspiration are widely acknowledged as primary contributors to SAP, it is important to note that both humoral and neurological changes following a stroke significantly impact the immune system [19, 31]. A leading theory is the concept of stroke-induced immunodepression syndrome (SIDS), which is thought to play a crucial role in the onset of SAP [32, 33]. The complex interplay between the immune and central nervous systems, which can be severely disrupted by an acute stroke, is critical to the prognosis of these patients [34, 35]. Such disruption may lead to the onset of SIDS, thereby increasing the susceptibility of individuals to pulmonary infections.

Fibrinogen, synthesized by the liver as an acute phase reactant, functions as a nonspecific marker of inflammation, with levels rising in response to inflammatory processes [17–19]. Notably, fibrinogen accumulates at sites of inflammation, thereby exacerbating the inflammatory response [19, 36]. It is significant that epithelial cells from various tissues, including those in the lungs, can also produce fibrinogen in response to inflammatory stimuli [37]. This local production of fibrinogen in the lungs may contribute to the observed association with SAP risk, potentially creating a pro-inflammatory environment that facilitates bacterial growth and impairs host defense mechanisms. In a previous study, fibrinogen levels were observed to be higher in patients who experienced hemorrhagic transformation (HT) following AIS compared to those without HT after intravenous thrombolysis [38]. This elevation may be attributed to the inflammatory response initiated by the onset of stroke. As the immune system responds to ischemic brain events, stroke-induced immunodepression may serve to protect the body from excessive inflammatory responses. However, this immunodepression simultaneously increases the risk of concurrent infections and adversely affects the prognosis of stroke patients [32, 39].

Our findings have significant clinical implications. The strong association between fibrinogen levels and SAP risk suggests that routine fibrinogen testing upon admission for AIS could enhance early identification of high-risk patients. This could potentially guide more targeted preventive strategies, such as intensive dysphagia screening or prophylactic antibiotic treatment in high-risk individuals. However, further research is needed to validate the efficacy of such approaches.

However, it is important to acknowledge several limitations in this study. Firstly, the relatively small sample size drawn from a single institution may lead to selection bias, emphasizing the necessity for broader, multicenter, and prospective studies to validate these findings. Secondly, the exclusive measurement of fibrinogen levels at the time of admission limits our understanding of their potential dynamic changes in relation to disease progression. Lastly, the applicability of our findings is restricted to patients without intracerebral hemorrhage, underscoring the critical need for future research to explore the role of fibrinogen as a predictive biomarker across all stroke subtypes. This would provide a more comprehensive understanding of its clinical relevance in stroke management.

## Conclusion

Prompt diagnosis and early intervention for SAP are imperative, as they significantly improve patient outcomes, increase survival rates, and reduce the caregiving and economic burdens associated with SAP. The current study reveals that fibrinogen levels have the highest predictive value for SAP in patients with AIS within a medical ward setting. Taken together, these findings suggest that fibrinogen could be effectively utilized as a screening tool for the early detection of SAP in AIS patients in clinical environments. However, further research is essential to more thoroughly understand the role of fibrinogen and its underlying mechanisms across various aspects of AIS.

## Data Availability

The datasets used and/or analysed in the current study are available from the corresponding authors upon reasonable request.

## Abbreviations

TC: Total cholesterol
TG: Triglyceride
HDL-C: High-density lipoprotein cholesterol
LDL-C: Low-density lipoprotein cholesterol
SAP: Stroke-associated pneumonia
AIS: Acute ischemic stroke
SIDS: Stroke-induced immunodepression syndrome
